# Physicochemical and Toxicological Properties of Particles Emitted from Scalmalloy During the LPBF Process

**DOI:** 10.3390/toxics13050398

**Published:** 2025-05-15

**Authors:** Nikoletta Sargioti, Leonidas Karavias, Leonidas Gargalis, Anna Karatza, Elias P. Koumoulos, Evangelia K. Karaxi

**Affiliations:** 1 Conify, P. Nikolaidi 23A, Agios Ioannis Rentis, 182 33 Athens, Greece; nsargioti@conify.gr (N.S.); lkaravias@conify.gr (L.K.); lgargalis@conify.gr (L.G.); 2 BioG3D, P.C., P. Nikolaidi 23A, Agios Ioannis Rentis, 182 33 Athens, Greece; akaratza@biog3d.gr; 3 IRES—Innovation in Research & Engineering Solutions, Silversquare Europe Square de Meeûs 35, 1000 Brussels, Belgium; epk@innovation-res.eu

**Keywords:** Scalmalloy, laser powder bed fusion (LPBF), particle morphology, cell viability, oxidative stress

## Abstract

This study investigates the physicochemical and toxicological properties of Scalmalloy powder emissions generated during Laser Powder Bed Fusion (LPBF), focusing on the impact of particle morphology, oxidation, and size distribution on biological responses. Scanning Electron Microscopy (SEM) and Energy Dispersive Spectroscopy (EDS) analyses revealed significant variations in particle characteristics, with the highest oxidation levels and irregular morphologies observed in exhaust-derived powders. In vitro cytotoxicity evaluations using A549 lung epithelial cells showed significant reductions in cell viability (~60 to 69%) and increased oxidative stress (*p* < 0.05) upon exposure to virgin sieved (<20 µm) and exhaust powder samples. Conversely, samples from the build plate, overflow, and dispenser exhibited high cell viability (>85%). Indirect exposure through media incubation resulted in minimal cytotoxicity, suggesting that metal dissolution plays a limited role in toxicity under the studied conditions. The findings highlight the influence of particle morphology and oxidation on cytotoxic responses and underscore the need for controlled powder handling to mitigate occupational exposure risks in LPBF environments.

## 1. Introduction

Additive Manufacturing (AM) has improved the manufacturing environment by enabling the creation of complex and highly customized components with minimal material waste and reduced production times. Unlike traditional subtractive manufacturing methods, which involve removing material from a solid block, AM builds components layer by layer directly from a digital model, allowing for greater design flexibility and complex geometries that would be challenging or impossible using conventional techniques [1]. Among AM techniques, Laser Powder Bed Fusion (LPBF) is widely used for fabricating high-density metal parts with excellent mechanical properties. Laser powder bed fusion spreads a few microns-thin layer of metal powder over a build platform and uses a high-energy laser to selectively melt and fuse the powder, repeating this process layer by layer until the component is complete. This technique offers high precision, the ability to process various metals and alloys, and the capability to produce intricate internal structures [2,3]. However, the rapid heating and cooling cycles in LPBF result in unique microstructural features, including a bimodal grain distribution with fine equiaxed and larger columnar grains. While these features enhance mechanical performance, they also introduce challenges such as porosity and defect formation [3]. The interaction between high-energy lasers and metal powders further influences the morphology and distribution of fine and ultrafine particles, which depend on parameters such as laser power, scan speed, and alloy composition.

The complex interactions between laser energy and metal powder in LPBF inevitably alter powder characteristics, leading to degradation influenced by process parameters and build topology. Factors such as melted volume fractions, part spacing, and fabrication heights significantly impact powder quality during the process [4]. Powder oxidation is primarily driven by environmental conditions, including temperature, exposure time, atmosphere composition, and build topology, which influence the extent of material degradation. LPBF systems use inert gas flow that removes process by-products from the process zone such as spatter to enable an undisturbed process [5]. Reducing the oxygen content in the build chamber is an efficient approach to prevent spatter from generating. Through multiple gas circulations, the equipment can decrease the oxygen level in the build chamber.

Spatter is an inevitable by-product of LPBF, directly impacting part quality by introducing metallurgical defects and degrading powder properties [6,7]. Spatter particles disrupt powder layers, leading to non-fusion defects [8,9]. They are categorized into hot droplet spatter, originating from melt pool instability due to vapor-induced recoil pressure, and cold powder spatter, driven by vapor-induced gas entrainment. Gasper et al. [10] classified spatter into seven groups based on size, morphology, oxide presence, and agglomeration. Laser energy loss due to spatter and welding fumes affects LPBF efficiency. While welding fumes minimally influence part porosity [11,12], spatter deposition in downstream areas increases porosity, as demonstrated by Ali et al. [13] and supported by Ladewig et al. [14] and Anwar and Pham [15]. Fine particles preferentially deposit during coating, while larger particles are displaced to the overflow. Spatter oxidation further impacts powder and part quality. Post-LPBF, oxidized spatter forms a surface oxide layer, reducing liquid metal wettability [16]. These oxidized particles require higher melting energy, leading to incomplete fusion and potential defects [17]. Additionally, redeposition of oxidized spatter into the melt pool can reverse Marangoni convection flow, exacerbating defects [18,19]. While spatter and oxidation-related defects are critical concerns in LPBF, the material-specific challenges of alloy compositions, such as evaporation-induced inhomogeneities and health risks from fine metal particle emissions, further complicate the process [20].

Aluminum alloys are particularly valued for their lightweight nature, strength, and versatility. However, key alloying elements such as magnesium and zinc tend to evaporate during the melting process, generating vapor plumes and condensates. These phenomena contribute to the formation of non-equilibrium phases and localized material inhomogeneities, which can significantly influence the mechanical integrity of fabricated components [21]. Beyond material integrity, the LPBF process also introduces important health and safety concerns due to the emission of fine and ultrafine metal particles [22]. The high-energy nature of LPBF, combined with the use of fine metal powders, leads to the formation of airborne particles, which pose inhalation and dermal exposure risks. Fine particles, particularly those under 10–20 µm, are more likely to penetrate deep into respiratory tissues [23,24,25], while fine particles (<1 µm) can enter via the bloodstream or pose a risk through dermal exposure by penetrating via hair follicles or compromised skin, potentially affecting other organs and leading to localized or systemic effect [26]. Larger particles, though less likely to reach alveolar regions, may still deposit in the upper respiratory tract and contribute to respiratory exposure, triggering localized inflammatory responses [27].

Investigations into alloys such as 316L stainless steel, Inconel, and other nickel- and cobalt-based powders have shown that emitted particles, during LPBF, exhibit high surface reactivity and bio-accessibility under physiological conditions, although limited ROS generation and no cytotoxicity were observed in the tested cell models [28]. Studies investigating the bioaccessibility of alloy constituents under physiological conditions have shown that metals such as nickel, cobalt, and chromium can be released from stainless and low-alloyed steels, with their extent influenced by surface oxide characteristics and corrosion resistance [29]. Nickel exposure is linked to allergic reactions, respiratory irritation, and at high levels, it is classified as a carcinogen. Cobalt can lead to respiratory issues, skin sensitization, and even cardiotoxic effects with prolonged exposure [30]. Chromium, depending on its oxidation state, poses significant risks, with hexavalent chromium (Cr6+) being a well-documented carcinogen and highly toxic to humans [31]. While these elements provide desirable mechanical and corrosion-resistant properties in LPBF alloys, their presence requires strict safety measures to mitigate exposure risks, particularly in powder form.

Recent findings suggest that aluminum powders used in AM processes, especially when reused, tend to accumulate oxygen due to repeated exposure to ambient air, leading to increased oxide formation on particle surfaces [32]. This oxidation alters surface chemistry, which may enhance the release of reactive oxygen species (ROS) and trigger inflammatory responses upon inhalation [33]. Studies on Al-based LPBF powders indicate that fine and oxidized particles can induce significant cytotoxic and pro-inflammatory effects in lung epithelial cells, potentially through oxidative stress-mediated pathways. Moreover, oxidation in aluminum powders has been shown to be strongly influenced by both powder handling and thermal exposure during printing. Repeated laser scanning cycles can significantly increase oxygen content, particularly in powders exposed to high heat near the scanning zones. Among aluminum-based alloys, AlSi10Mg [34,35] and AM-grade Scalmalloy [36,37] are gaining considerable attention in LPBF applications due to their lightweight properties and mechanical performance. In five production cycles, the oxygen content of AlSi10Mg powders is reported to increase by 120 ppm due to laser-induced heating, whereas powders that were only recycled (without heat exposure) showed a much smaller oxygen increase of 10 ppm. These findings suggest that thermal history plays a critical role in powder oxidation, which may, in turn, influence bioavailability of metal ions such as aluminum, magnesium, or scandium, and cytotoxic potential [38]. The bio-accessibility of aluminum ions under physiological conditions has also been linked to increased cytokine release and inflammatory signaling, highlighting the need for further assessments on aluminum powder toxicity [39]. Additionally, spatter formation during LPBF has been identified as a key contributor to oxidation, with highly oxidized spatter particles being reintroduced into the powder supply, further increasing oxygen content over multiple reuse cycles. These oxidized particles, often smaller in size, have a greater surface-area-to-volume ratio, making them more prone to interacting with biological tissues and potentially exacerbating oxidative stress responses [38]. Post-processing tasks, such as powder handling, sieving, and part cleaning, have been identified as major sources of exposure, often overshadowing emissions during the printing process itself [24]. These findings identify the importance of implementing effective exposure control measures, including proper ventilation, personal protective equipment, and process monitoring, to safeguard workers in LPBF environments employing such systems.

The biological impact of these emissions is further influenced by particle characteristics, such as shape and surface irregularities, which play a critical role in determining their interactions with tissues. Irregularly shaped particles, often formed due to spatter during LPBF, can exhibit sharp edges, leading to mechanical irritation of tissues. These irregularities also increase their surface area, enhancing chemical reactivity and potential oxidative stress. In contrast, spherical particles, typically produced via evaporation–condensation mechanisms, have smoother surfaces but still pose risks due to their high surface-to-volume ratios [40]. Surface area is related to reactivity, as ultrafine particles with a high surface-area-to-mass ratio can generate ROS when interacting with biological fluids. This increased reactivity can result in oxidative stress and cellular damage, highlighting the critical importance of understanding surface-area-related toxicity [41].

Scalmalloy, an aluminum-based alloy composed of aluminum (Al), magnesium (Mg), and scandium (Sc), is designed to offer high performance with relatively low toxicity [21]. Magnesium, while generally considered low in toxicity [42], may still pose risks under certain conditions, such as respiratory exposure to fine dust in occupational settings. Agglomeration is another key factor that influences the toxicity of particles. Small particles often cluster into larger agglomerates during or after emission. These agglomerates can alter aerodynamic behavior, affecting deposition in the respiratory tract. While larger agglomerates may settle in the upper respiratory system, they can disaggregate into finer components, increasing the risk of deep penetration and systemic distribution [43]. Finally, the porosity and surface roughness of particles can influence their dissolution rates, ability to adsorb proteins, and interaction with cellular membranes, further modulating their toxicological behavior [44]. However, the toxicological effects of its constituent elements, particularly scandium (Sc), have not been extensively studied, especially in fine particulate form. Kawai et al. [45] have shown that low concentrations of Sc can stimulate antibiotic production in certain Streptomyces cultures. Furthermore, studies have demonstrated the accumulation of scandium in tissues such as the liver, spleen, and bone, with some evidence of biological effects on various cancer cell lines, including osteosarcoma.

Some studies suggest that Sc-containing compounds may influence biological systems, such as enhancing antibiotic production in certain microbial cultures, while prolonged inhalation exposure in rare-earth mining environments has been associated with potential respiratory risks, including the possibility of lung embolism [46]. Nonetheless, comprehensive data on the long-term toxicity and environmental impact of scandium remain limited and require further investigation.

The present study aims, therefore, to bridge the knowledge gap by evaluating the physicochemical and toxicological properties of Scalmalloy powders generated during LPBF processes. The analysis includes both virgin and used powders, with a focus on understanding how processing conditions influence particle morphology, composition, and reactivity. By investigating the interactions of these particles with biological systems, this research seeks to provide an understanding of the health implications associated with Scalmalloy in additive manufacturing contexts.

## 2. Materials and Methods

### 2.1. Sampling and Preparation of Scalmalloy Powders

The AM process involved manually loading powder into a PBF system (INTECH, SF1 iFusion150, Intech Additive Solutions Ltd., Bangalore, India), conducting a 44-h print job under an argon atmosphere, and manually removing the printed build by performing de-powdering within a controlled environment. Additionally, the printer is cleaned after use, and the unused powder is sieved for reuse.

**Figure 1:**
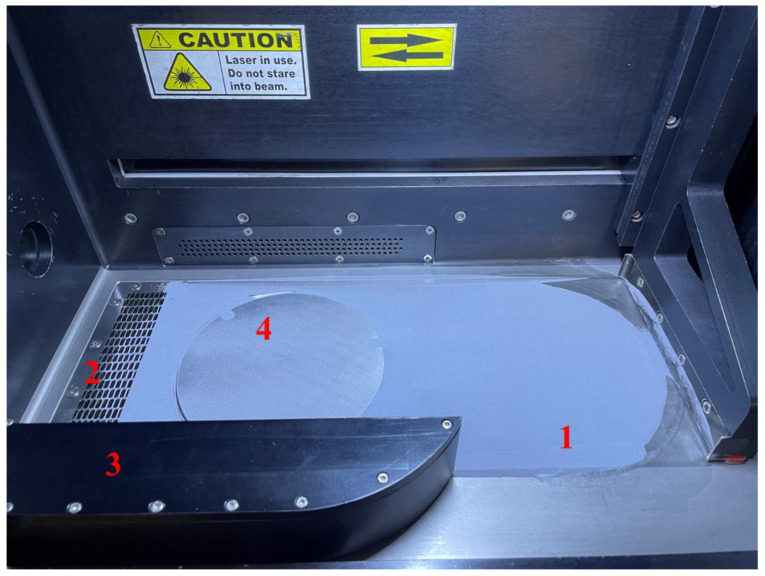
Sampling locations for Scalmalloy powders within the LPBF system, indicated by numbered labels. The dispenser (1) represents the as-received virgin powder (feed region) prior to processing. The overflow (2), exhaust (3), and build plate (4) samples were collected 44 h after the completion of the printing process to assess changes due to thermal exposure.

To assess the quality of Scalmalloy powder, a sampling methodology was implemented across different locations within the build chamber of the LPBF machine. A schematic of these locations is shown in Figure 1. Sampling included the dispenser sample (virgin powder (number 1)) before any processing or sieving, and sieved virgin powder (<20 µm) to represent inhalable fractions. Virgin powder was represented by the as-received material, while the <20 µm virgin powder sample was subjected to repetitive sieving cycles and powder handling using a lab-scale tabletop sieve shaker (TA 005, Ortoalresa, Madrid, Spain), equipped with stainless-steel sieves (AISI 316 mesh), in an open oxidative atmosphere with up to 50% relative humidity. The sieving was performed using 50 g of powder for 15 min in accordance with ASTM B214-22 [47]. Furthermore, used powder was collected from the overflow (number 2), exhaust (number 3), and build plate (number 4) after the 44-h printing process (Figure 1). Within the build plate and the exhaust, the majority of spatter and highly oxidized particles were expected due to the inert gas flow. The inert gas directs agglomerated and spatter particles to the exhaust, which is anticipated to yield the most deteriorated powder sample in terms of chemical composition (e.g., evaporation of Mg, high oxygen content on particle surfaces) and morphology (e.g., deformed particles, reduced sphericity from heat exposure). This methodology allowed a comprehensive characterization of the powders’ properties and assessment of changes due to thermal exposure during the process. All samples were transferred into sterile tubes to prevent contamination, preserving the integrity of the material for subsequent toxicity evaluations. This approach ensured that external factors did not influence the assessment of the powders’ physicochemical and biological properties.

### 2.2. Scanning Electron Microscopy–Electron Dispersive Spectroscopy (SEM-EDS)

**Figure 2:**
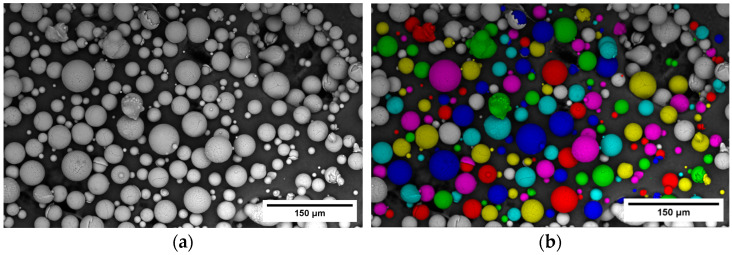
Indicative example of SEM images before (**a**) and after (**b**) the identification of particles with Phenom ParticleMetric software, V1.2.2.0.

Gas-atomized Scalmalloy^®®^ (APWORKS GmbH, Taufkirchen, Germany) metal powder was used as feedstock with chemical composition presented in Table 1. The term ‘Al balance’ indicates that aluminum is the primary element in Scalmalloy, and its content adjusts to balance the total composition after accounting for the listed alloying elements. The particle size distribution (PSD), shape, and morphology of the Scalmalloy powder samples were determined using Scanning Electron Microscopy (SEM) (PhenomProX, Thermo Fisher Scientific Inc., Waltham, MA, USA) and an add-on image analysis software (Phenom ParticleMetric, V1.2.2.0) from ThermoFisher Scientific, Waltham, MA, USA. The software highlights the detected particles according to user-defined settings, which can be adjusted to enhance particle detection and improve the identification of irregular and agglomerated particles (e.g., Figure 2). To ensure statistically relevant results, three measurements comprising 120 SEM micrographs per sample were acquired through Automated Image Mapping and analyzed, with each micrograph captured at a 500 μm Field of View (FOV). To assess the shape and morphology of powder particles, the shape descriptor of circularity and convexity was calculated based on the samples’ volumetric distribution.

Furthermore, the chemical composition of each powder sample was analyzed using electron dispersive spectroscopy (EDS) (PhenomProX, Thermo Fisher Scientific Inc., Waltham, MA, USA), integrated with the SEM instrument, ensuring a measurement accuracy of 0.1%. Area EDS chemical analysis was performed on each powder using a 20 kV acceleration voltage to ensure optimal compositional analysis. The analysis was carried out at the lowest possible magnification (×880) to capture as many particles as possible. Five area EDS measurements were taken for each sample, covering the entire surface to ensure statistically relevant data. The chemical composition measurements were performed to evaluate any variations in composition between the samples. Additionally, localized EDS elemental mapping measurements were conducted on oxidized particles of the exhaust powder sample to evaluate compositional variations.

### 2.3. Cell Lines and Cell Culture

A549 cells were cultured in complete Dulbecco’s Modified Eagle Medium (DMEM, BIOSERA, Cholet, France) with high glucose, supplemented with 10% fetal bovine serum (FBS, BIOSERA, Cholet, France) and 1% penicillin/streptomycin (BIOSERA, Cholet, France). This medium provided a physiologically relevant in vitro environment for evaluating the biological response to Scalmalloy particle exposure via inhalation. For cell recovery, frozen vials were thawed by gentle agitation in a 37 °C water bath and subsequently disinfected with 70% ethanol (Sigma-Aldrich, St. Louis, MO, USA). All subsequent steps were performed under aseptic conditions. The thawed cell suspension was transferred to a 15 mL centrifuge tube containing 9 mL complete culture medium and centrifuged at 125× *g* for 5 min. After centrifugation, the supernatant was removed, and the resulting cell pellet was resuspended in complete culture medium by gently pipetting the cells up and down three times to achieve a uniform cell suspension. Culture vessels containing complete growth medium were pre-incubated at 37 °C for 15 min to equilibrate the pH (7.0 to 7.6). The culture suspension was then added, and cultures were maintained at 37 °C in a humidified incubator with 5% CO_2_ in air. Once the cultures reached approximately 90% confluency, subculturing was performed. The culture medium was discarded, and the cell layer was rinsed with phosphate-buffered saline (PBS, BIOSERA, Cholet, France) (1× solution) to remove residual serum. Trypsin-EDTA solution(BIOSERA, Cholet, France) (2–3 mL) was added, and cells were monitored under an inverted microscope until detachment, typically within 5–15 min. For difficult-to-detach cells, incubation at 37 °C was used to facilitate detachment. Complete medium (6–8 mL) was added to neutralize the trypsin, and the cell suspension was gently aspirated. Appropriate volumes were transferred to new culture vessels and incubated at 37 °C. 

### 2.4. Exposure Conditions

Cells are exposed to various powder per volume fractions of Scalmalloy powder samples (dose response relationship) for different time periods. Based on the literature, indicative amounts of the powder per volume to be used experimentally are 0, 5, 10, 20, 30, 40, 50, and 60 μg/mL [32]. Given that metals require time to oxidize, powders were incubated alone in the cell culture media (without cells) for an extended period of 48 h in a concentration of 100 μg/mL. This step was crucial to assess the potential release of metal ions due to oxidation, which could influence cellular responses. After incubation, the samples were vortexed and centrifuged at 3357× *g* for 30 min. No sonication was applied in order to preserve particle morphology and surface characteristics. Without disturbing the pellet (undissolved particles), 500 μL supernatant, considered as the “release fraction”, was collected and subsequently used to treat the cells [48]. This method allows differentiation between direct particle effects and potential toxicity caused by released metal ions.

### 2.5. Cell Viability

Cell viability was assessed using the MTT assay (3-(4,5-dimethylthiazol-2-yl)-2,5-diphenyltetrazolium bromide, Sigma-Aldrich, St. Louis, MO, USA), which measures mitochondrial activity by quantifying the reduction of MTT to formazan by metabolically active cells. A549 cells were seeded in triplicate at 250 μL per well in clear, flat-bottom 48-well plates. After exposure to Scalmalloy powder, cells were incubated at 37 °C for 48 h. To minimize interference with the metal particles, media-only wells and untreated control cells were included as reference. Following incubation, the medium was removed without disturbing the formed formazan crystals. A quantity of 200 µL of dimethyl sulfoxide (DMSO, PAN-Biotech GmbH, Aidenbach, Germany) was used as a solubilization solution. The plates were covered with foil and placed on an orbital shaker for 15 min to ensure complete solubilization. Absorbance was measured at 590 nm using a FLUOstar^®®^ Omega plate reader (BMG Labtech, Ortenberg, Germany). The absorbance values were normalised to untreated control cells (set as 100%) to determine cell viability. The intensity of the resulting purple color, corresponding to the amount of solubilized formazan, is directly proportional to the number of metabolically active cells.

### 2.6. Oxidative Stress Assays

Intracellular reactive oxygen species (ROS) generation upon exposure to Scalmalloy powder was assessed using the ROS Detection Cell-Based Assay Kit (DCFDA, Item No. 601520, Cayman Chemical, Ann Arbor, MI, USA). This assay is based on the oxidation of 2′,7′-dichlorofluorescin diacetate (DCFDA), a non-fluorescent probe that, upon cellular uptake and deacetylation, is converted into 2′,7′-dichlorofluorescin (DCFH). In the presence of ROS, DCFH is further oxidized to 2′,7′-dichlorofluorescein (DCF), a highly fluorescent compound that can be detected using a fluorescence plate reader.

A549 cells were seeded in clear flat-bottom 48-well plates at a density of 2 × 10^4^ cells per well and allowed to attach overnight at 37 °C in a humidified 5% CO_2_ incubator. After treatment with Scalmalloy powder for 48 h at concentrations of 5 μg/mL to 60 μg/mL for direct exposure, and 100 μg/mL for indirect exposure, cells were washed with serum-free DMEM and incubated with 25 μM DCFDA in Hank’s Balanced Salt Solution (HBSS) for 45 min at 37 °C in the dark. Following incubation, excess DCFDA was removed, and fresh HBSS was added before measuring fluorescence at Ex/Em = 485/535 nm using a FLUOstar^®®^ Omega plate reader (BMG Labtech, Ortenberg, Germany).

Fluorescence intensity was normalized to untreated control cells (set as 100%) to quantify ROS production. A positive control (e.g., tert-butyl hydroperoxide, TBHP, 50 μM) was included to verify assay performance.

### 2.7. Cellular Morphology via SEM

To evaluate cell morphology after powder exposure, A549 cells were seeded on sterile glass coverslips and treated with 60 μΜ of Scalmalloy powder for 48 h. Following exposure, cells were fixed using 4% paraformaldehyde (PFA) in Phosphate-Buffered Saline (PBS) for 15 min at room temperature. Cells were then washed three times with PBS and dehydrated using a graded ethanol series (30%, 50%, 70%, 90%, and 100%, 5 min each). For optimal SEM imaging, after the last ethanol wash, cells were immersed in Hexamethyldisilazane (HMDS, Sigma-Aldrich, St. Louis, MO, USA) for 5–10 min to achieve gentle drying and minimize structural shrinkage, then air-dried in a fume hood. Dried coverslips were mounted on SEM stubs using conductive tape. Cell morphology was examined using a scanning electron microscope at an accelerating voltage of 5 kV with a secondary electron detector.

## 3. Results

### 3.1. Morphology and Chemical Analysis of Scalmalloy Powder Samples 

**Figure 3:**
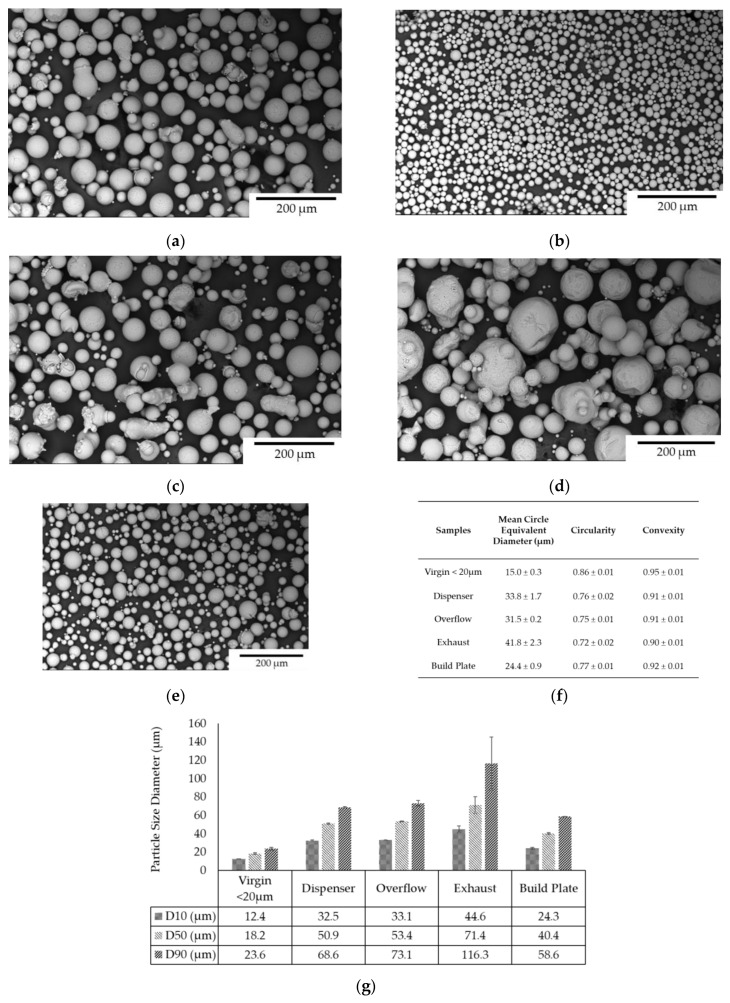
SEM images of Scalmalloy powder showing particle morphology across various samples. (**a**) Dispenser sample, (**b**) Virgin powder (<20 μm), (**c**) Overflow sample, (**d**) Exhaust sample, and (**e**) Build plate sample, after 44 h of printing. (**f**) Table summarizing the mean circle equivalent diameters along with circularity and convexity values for each sample. (**g**) Bar graph illustrating the D10, D50, and D90 particle size distributions for each sample.

The morphology and composition of Scalmalloy powder samples were analyzed using SEM-EDS. The SEM images (Figure 3) indicate that the particles across all samples exhibited a predominantly spherical morphology, with circularity and convexity values reaching up to 0.87 and 0.95, respectively, signifying a high degree of particle roundness. However, variations in PSD, shape, and oxidation levels were observed, potentially influenced by the powder’s exposure to different processing environments.

Post-printing (~44 h), the Dispenser sample (Figure 3a, see table in Figure 3f) exhibited a mean diameter of 33.8 μm with a circularity of 0.76 and a convexity of 0.91. The overflow sample (Figure 3c,f,g) had a comparable mean diameter of 31.5 μm, a circularity of 0.75, and 0.91 convexity, but exhibited a broader size distribution with D90 reaching 73.1 μm. The overflow sample contained more elongated and irregular particles, possibly due to its exposure to printing conditions that promote sintering or particle fusion. The exhaust sample (Figure 3d,f,g) showed the most significant deviation from the other samples, presenting the largest average diameter (41.8 μm) with a wider PSD (D10 = 44.6 μm, D50 = 71.4 μm, and D90 = 116.3 μm) and the lowest circularity and convexity values (0.72 and 0.90, respectively). The build plate sample (Figure 3e,f) displayed an average diameter of 24.4 μm with a higher circularity of 0.77 compared to the exhaust sample, indicating a more uniform and spherical morphology. The narrower size distribution was reflected in D10, D50, and D90 values of 24.3 μm, 40.4 μm, and 58.6 μm, respectively. The virgin powder upon sieving (<20 μm) (Figure 3b,f) showed the smallest average diameter of 15.0 μm, as expected, with circularity and convexity values of 0.86 and 0.95, respectively. Its PSD, with D10, D50, and D90 values of 12.4 μm, 18.2 μm, and 23.6 μm, respectively, was relatively narrow compared to the other powder samples.

**Figure 4:**
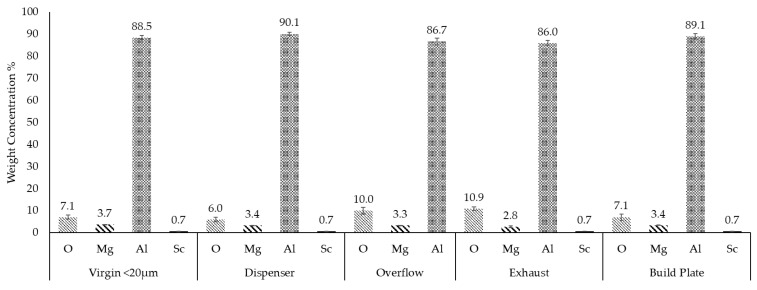
Area EDS chemical analysis of the samples.

The oxygen concentration varied across samples, with the highest levels detected in the exhaust and overflow powders, measuring 10.9 wt.% and 10.0 wt.%, respectively (Figure 4). These findings suggest that finer particles and those exposed to prolonged processing are more susceptible to oxidation, likely due to increased surface area and thermal exposure. In contrast, the dispenser sample exhibited the lowest oxygen content (6%), indicating minimal oxidation and better preservation of powder quality within the system. The virgin (<20 μm) and build plate powder samples showed similar oxygen weight concentrations of 7.1% comparable to the dispenser powder sample.

The aluminum content remained dominant (~85 to 90 wt.%), as expected for Scalmalloy composition, with minimal changes across the different samples (Figure 4). These findings highlight the influence of powder processing conditions on particle morphology and oxidation. While the dispenser powder maintained good spherical morphology and low oxidation levels, the exhaust and overflow samples exhibited higher oxidation and broader PSD, indicating potential degradation and contamination risks. The oxygen content in fine (<20 µm) virgin powder also suggests that sieving contributes to oxidation likelihood, likely due to exposure to an open, oxidative atmosphere with up to 50% relative humidity, highlighting the need for controlled storage and handling conditions.

### 3.2. Cell Viability upon Treatment with the Scalmalloy Powder Samples in Various Concentrations

**Figure 5:**
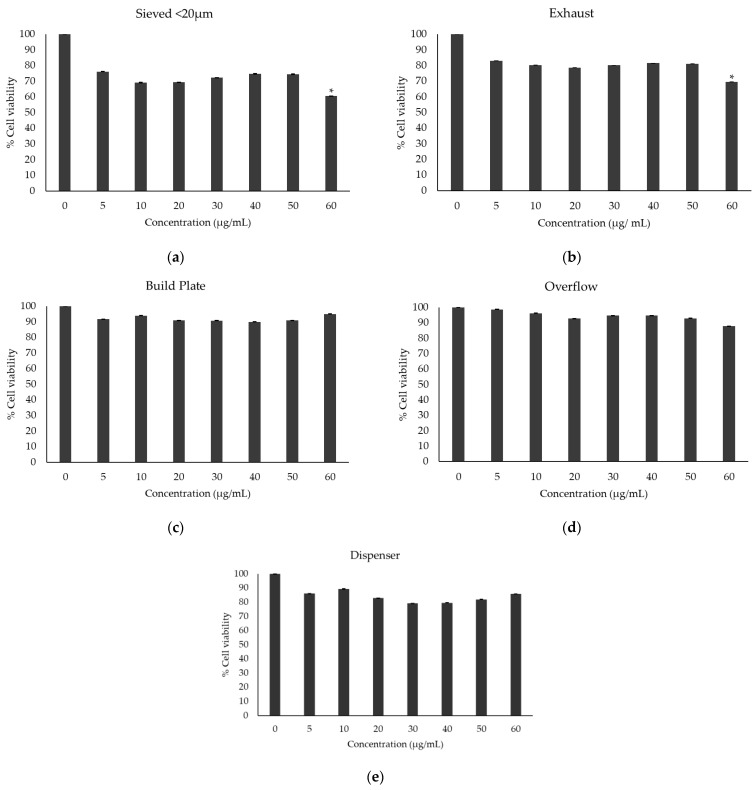
Cell viability upon treatment with the Scalmalloy powder samples: (**a**) Sieved powder (<20 μm) sample, (**b**) Exhaust sample, (**c**) Build plate sample, (**d**) Overflow sample, and (**e**) Dispenser sample. The results are expressed as percentage means ± standard deviation in comparison to the untreated control group. Statistical analyses were conducted using One-Way ANOVA and T-tests, with *p*-values considered significant at <0.05.

The MTT assay was conducted to evaluate the cytotoxic effects of Scalmalloy powder samples on A549 lung epithelial cells following a 48-h exposure across a range of concentrations. The results indicate that most samples did not induce significant cytotoxicity (Figure 5). However, the exhaust-derived powder at the highest tested concentration (60 μg/mL) caused a statistically significant reduction in cell viability (*p*-value < 0.05) (Figure 5b). Notably, the sieved powder < 20 μm resulted in cell viability below 80% at all tested concentrations with statistical significance (*p*-value < 0.05) (Figure 5a). The reduction in cell viability observed in the exhaust and sieved sample < 20 μm is likely linked to particle morphology and reduced particle size, respectively, as identified through SEM analysis. The cell viability analysis for the build plate, overflow, and dispenser samples did not reveal significant cytotoxic effects even for the highest concentration tested, in contrast to the abovementioned cases.

**Figure 6:**
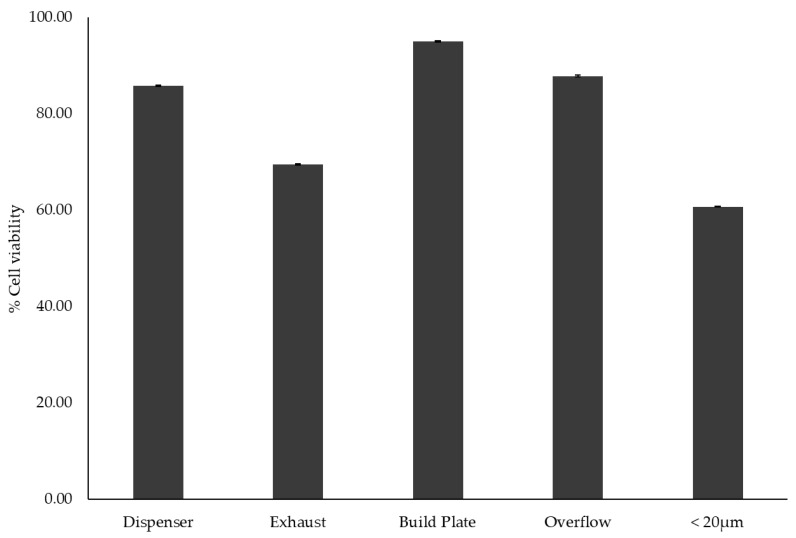
Cell viability rates in the 60 μg/mL concentration, indicating the highest viability for the sample obtained from the build plate and the lowest viability for the sample that was sieved under 20 μm.

Comparative analysis of cell viability at 60 μg/mL across all samples presented notable differences, as shown in Figure 6. The build plate sample exhibited the highest viability (~94%), followed by the overflow and dispenser samples (~87% and 85%, respectively). The exhaust sample demonstrated a slightly lower viability (~69%), while the 20 μm fraction of virgin powder had the lowest cell viability (~60%). These variations suggest that powder processing conditions influence cytotoxicity, likely due to differences in particle size distribution and surface morphology. The consistently higher cell viability observed in the build plate, overflow, and dispenser samples suggests that these powders retain relatively favorable physicochemical properties for biological compatibility.

The exhaust sample, which showed a pronounced decrease in cell viability (~69% at 60 μg/mL), corresponds to the sample with the highest oxygen concentration, as previously identified in the EDS analysis (Figure 4). This supports the hypothesis that oxidation plays a key role in cytotoxicity, potentially through the generation of ROS or alterations in particle reactivity. The virgin < 20 μm sample, despite being freshly sieved, exhibited the lowest cell viability (~60% at 60 μg/mL), likely due to its finer particle size, which increases surface area and enhances potential cellular interactions.

### 3.3. Oxidative Stress upon Treatment with the Powders in Various Concentrations

ROS generation was assessed to evaluate oxidative stress in A549 lung epithelial cells following a 48-h exposure to different Scalmalloy powder samples, with experiments carried out in triplicate. Oxidative stress levels varied between powder samples, with the highest ROS levels observed at 60 μg/mL in sieved < 20 μm and exhaust samples.

**Figure 7:**
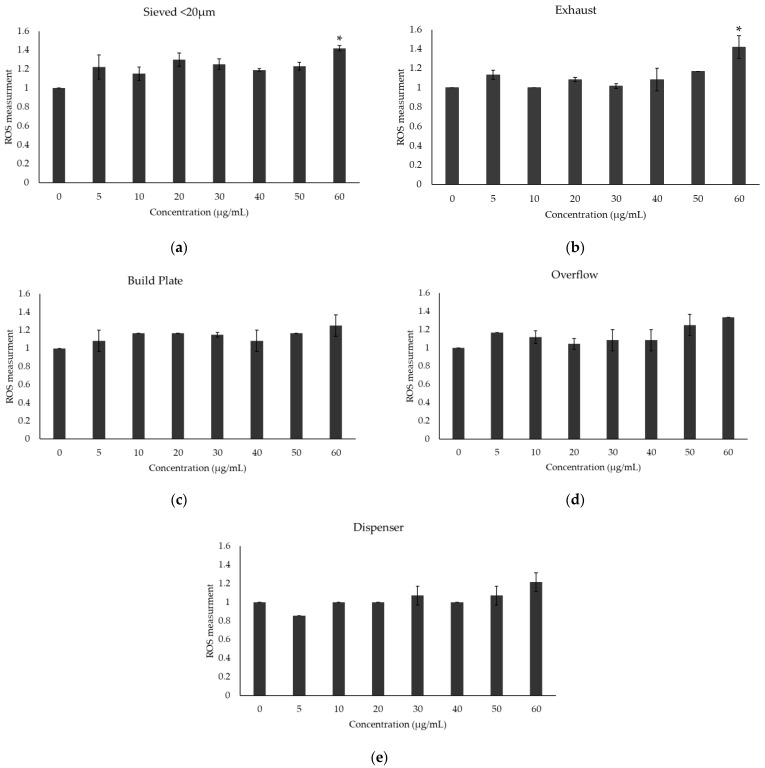
Oxidative stress upon treatment with Scalmalloy powder samples: (**a**) Sieved powder (<20 μm) sample, (**b**) Exhaust sample, (**c**) Build plate sample, (**d**) Overflow sample, and (**e**) Dispenser sample. Results are presented as mean percentages ± standard deviation relative to the untreated control group. Statistical analyses were performed using One-Way ANOVA and *t*-tests, with a significance threshold set at *p* < 0.05.

The virgin < 20 μm sample (Figure 7a) exhibited a modest but statistically significant (*p* < 0.05) increase in ROS levels at the highest concentration (60 μg/mL), suggesting that finer particles may induce oxidative stress due to their larger surface area and higher reactivity. Similarly, the exhaust sample (Figure 7b) demonstrated a significant (*p* < 0.05) ROS increase at 60 μg/mL, aligning with its previously observed cytotoxic effects and high oxidation levels. This suggests that powder collected from the exhaust area exhibits ROS-inducing potential due to the presence of oxidized metal surfaces, such as aluminum or magnesium oxides, or surface modifications that enhance oxidative stress responses in cells. In contrast, the build plate sample (Figure 7c) maintained relatively stable ROS levels across all concentrations, indicating minimal oxidative stress induction. This trend was also observed in the overflow (Figure 7d) and dispenser (Figure 7e) samples, which exhibited consistently low ROS levels, suggesting that these powders have minimal oxidative reactivity. The results imply that powders subjected to extensive oxidation, such as the exhaust sample, or those with smaller particle sizes, such as the virgin < 20 μm fraction, are more prone to inducing oxidative stress in biological systems. Overall, these findings suggest that oxidative stress responses vary depending on powder processing and collection conditions. The elevated ROS levels in the exhaust and virgin < 20 μm samples underscore the potential impact of oxidation and particle size and morphology on cellular responses.

### 3.4. Cell Viability and Oxidative Stress upon Treatment with the Aqueous Extracts Post-Incubation with the Scalmalloy Powder Samples

**Figure 8:**
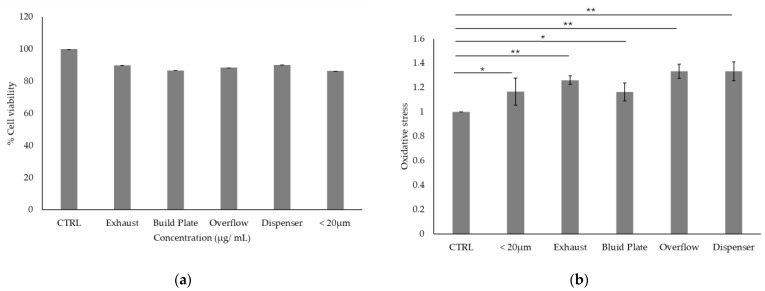
(**a**) % Cell viability, and (**b**) oxidative stress upon treatment to the media post-exposure with the Scalmalloy powder samples. Results are presented as mean percentages ± standard deviation relative to the untreated control group. Statistical analyses were performed using One-Way ANOVA and *t*-tests, * *p* < 0.05, ** *p* < 0.01.

As indicated in Figure 8a, cell viability remained high across all conditions, with no significant reduction compared to the control (CTRL), indicating that there is no significant release/leaching of toxic soluble factors from the powders into the media to induce acute cytotoxicity.

Regarding oxidative stress levels (Figure 8b), all Scalmalloy powder samples showed statistically significant increases compared to the control. The <20 μm sieved powder and overflow samples demonstrated the strongest effects (*p* < 0.01), while the exhaust, build plate, and dispenser samples also showed significant increases (*p* < 0.05). Cell viability remained high across all treatment groups, with no statistically significant reductions compared to the control. Nevertheless, all samples maintained viability above 85%, suggesting minimal cytotoxic effects under the tested conditions. While all powder samples induced statistically significant increases in oxidative stress compared to the control, these effects were moderate and did not correspond to reductions in cell viability. This suggests that, despite some ROS generation likely due to the leaching of metal ions, indirect exposure to Scalmalloy powders does not lead to pronounced cytotoxic effects under the tested conditions.

### 3.5. Cell Morphology Evaluation upon Treatment with the Scalmalloy Powders via SEM

**Figure 9:**
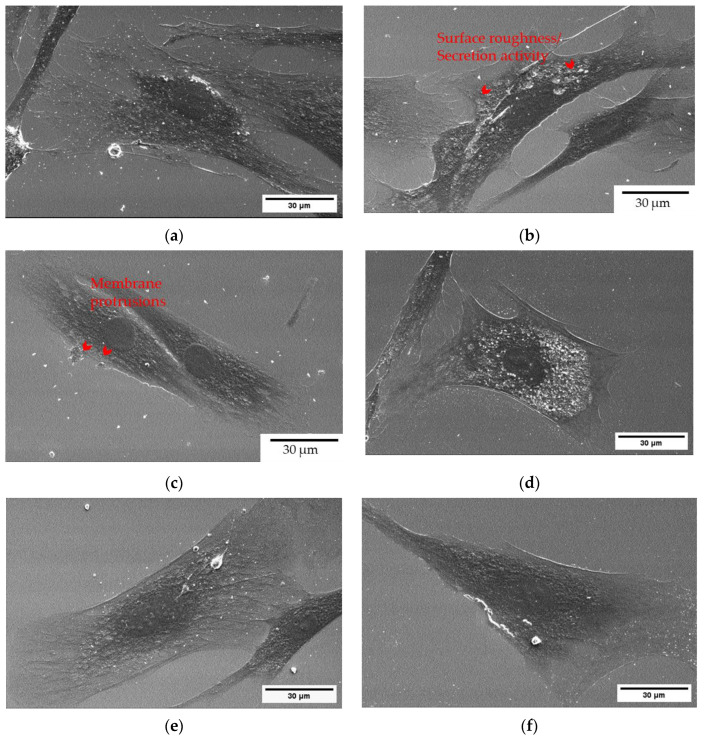
Morphology evaluation of the A549 cells upon treatment with the Scalmalloy powder samples. Representative images of the cell morphology obtained via SEM upon treatment with sample powders: (**a**) Untreated cells, (**b**) virgin powder < 20 μm, (**c**) exhaust, (**d**) build plate, (**e**) overflow, and (**f**) dispenser. Scale bar corresponds to 30 μm.

Cells were treated with 60 μg/mL of Scalmalloy powder samples for 48 h, following the protocol described in the Section 2. The SEM images in Figure 9 present morphological differences in the treated cells, depending on the source of the powder. The observed differences highlight the impact of specific powder fractions on cellular integrity and structural organization.

Control cells exhibited a normal, well-spread morphology with an intact cytoskeletal structure. The surface appeared smooth and uniform, with no visible membrane irregularities or damage, indicating a healthy cellular environment. In contrast, cells treated with virgin < 20 μm powder, while relatively well-preserved, exhibited increased surface roughness, as highlighted by the red arrows, suggesting a mild stress response. The changes were less pronounced than those observed in the exhaust powder-treated cells, but minor cytoskeletal alterations and localized irregularities were noted. Significant morphological alterations were observed in cells treated with exhaust powder, including irregular cell shapes, disrupted membrane structures, and smaller protrusions across the surface. Red arrows indicate regions of membrane degradation and cellular stress, suggesting that exhaust powder exposure induces higher cytotoxic effects compared to other samples. Cells treated with build plate powder displayed morphological features similar to the control group (CTRL), with no apparent alterations in spreading or surface structure. This suggests that build plate powder exposure does not significantly impact cell morphology. Similarly, cells treated with overflow powder maintained a well-spread morphology, comparable to the untreated control cells, with no visible membrane disruptions or stress markers, indicating minimal cytotoxic effects. While maintaining an intact morphology, cells treated with dispenser powder exhibited morphological changes, including slightly reduced spreading and minor surface irregularities. These features may indicate localized stress responses, although to a much lesser extent than seen in exhaust powder-treated cells.

These observations align with prior findings on oxidative stress and cell viability, reinforcing the understanding that particle source and oxidation levels influence cytotoxic responses. Exhaust powder exposure had the most pronounced impact, leading to cellular stress and surface degradation. Virgin < 20 μm powder induced moderate effects, with increased surface roughness but less disruption than the exhaust sample. Build plate and overflow powders had negligible effects, with treated cells displaying morphologies similar to the control group. Dispenser powder exposure led to mild changes, such as less spreading and minor surface alterations.

## 4. Discussion

This study provides an evaluation of the physicochemical and toxicological properties of Scalmalloy powders processed via LPBF, highlighting the influence of particle morphology, size distribution, and oxidation levels on cellular responses. The findings demonstrate that powder sampling location and particle size upon sieving (e.g., <20 μm) impact cytotoxicity and oxidative stress, with finer particles and morphologically altered particles exhibiting higher biological reactivity.

In the respiratory system, the bronchial epithelium serves as a protective mechanism for the lungs by maintaining stability and functioning as a mechanical barrier. Nonetheless, the secretion of cytokines and other external stimuli can lead to both acute and chronic responses during this process. The functional unit of human bronchial epithelial cells is critical, given the significant impact of these cells on biological stress responses.

Oxidative stress, widely recognized, is characterized by an imbalance between reactive species within the cell and the antioxidant defenses that neutralize them. Metals are integral to the human body, particularly in their role as ions within metalloproteins. Essential metals such as calcium, iron, and zinc are vital for numerous physiological functions, although their interactions within biological networks remain complex and incompletely understood. An overload or imbalance of metal ion concentrations can substantially impair cellular function and differentiation. Metal ions are crucial nutrients for host homeostasis and participate in many physiological processes; however, excessive or insufficient levels of metal ions may result in cellular dysfunction and death [49].

When ROS levels increase, cells respond by enhancing the expression of antioxidant defenses. However, if these defenses are insufficient, oxidative damage to proteins and nucleic acids can occur, compromising cell function and survival. Elevated ROS levels cause changes in the redox status of the cell, initiating inflammation and apoptosis. Oxidative stress can also result in various forms of oxidative DNA damage, such as strand breaks and base modifications. If these lesions remain unrepaired or repair mechanisms fail, genotoxicity may occur, leading to permanent DNA alterations. Although the mechanisms underlying the oxidant generation capacity of particulate matter after exposure of cells and tissues are not fully understood, it is known that cell exposure to particulate matter is associated with activation of NADPH oxidases, which are enzymes that generate O^2−^. Studies related to A549 cell toxicity have demonstrated that particulate matter water-soluble extracts, transition metals, and organic components can induce cytotoxicity, including reducing cell activity and increasing inflammatory responses and cytokine gene expression. Additionally, damage to the cell membrane and mitochondrial ultrastructure is a crucial aspect of cytotoxicity [50,51].

High particle concentration values are not always associated with high toxicity, because the chemical composition of the particulate itself can heavily influence the cytotoxic outcome [52]. The chemical composition, particle size, temperature, and many other properties influence the propensity for metallic particulates to disperse into ionic constituents. The relationship between chemical composition and the biological effects of particles has been explored, and metals (e.g., As, Pb, Fe, and Al) were found to be one of the major factors causing inflammation [51,53]. Transition metals (i.e., Al, Fe, Mn, Pb, Zn) might be involved in a time- and/or dose-dependent toxicity relying on inflammatory processes [54,55].

Under normal conditions, the concentration of metal ions is maintained in dynamic balance through the regulation of various transporter proteins and storage mechanisms. However, exposure to radiation, tumors, or accidental injuries can lead to an elevated intracellular concentration of metal ions, resulting in ionic overloading. This can cause an abnormal accumulation of metal ions that bind to inappropriate receptor sites, leading to signaling disorders and producing toxic effects [56,57,58,59]. In recent years, increasing evidence has suggested that Fe^3+^ and Cu^2+^ overloading could cause a toxic stress response, ultimately leading to cell death [60,61]. Transition metals like nickel, zinc, copper, and manganese cause inflammation and genetic toxicity. They induce reactive oxygen species (ROS), which damage cell membranes, proteins, and DNA, potentially leading to cell death. Water-soluble components activate ROS, release inflammatory cytokines, and are linked to cell apoptosis and genetic toxicity [62,63].

Metal oxide and metal alloy-based micron-sized particles tend to exhibit a lower degree of stability and are more susceptible to dissolution and ion release when introduced to a biological milieu, leading to reactive oxygen species (ROS) production and oxidative stress to cells. Continuous exposure to particulate-matter-induced ROS can result in inflammation. It is speculated that the toxicity is related to the soluble fraction rather than the insoluble particles, and that the soluble metal ions have the potential to cause pulmonary epithelial cell (A549) injury as well as an inflammatory response. Moreover, according to another study, the water-soluble fraction of particulate matter is responsible for the ROS stimulation in pulmonary epithelial cell (A549), especially in the early exposure stage [53].

The particle size distribution (PSD) and morphology varied among the powder fractions. Build plate, dispenser, overflow, and virgin < 20 μm powders showed relatively uniform and spherical particle shapes, while exhaust samples displayed more irregular and agglomerated particles. Circularity is a crucial parameter that reflects the deviation of particles from an ideal spherical shape [64]. The highest circularity value (0.86) was measured in the virgin < 20 μm powder sample. The build plate and dispenser samples exhibited similar circularity values (0.77 and 0.76, respectively), while the overflow sample also demonstrated comparable circularity, reaching 0.75. In contrast, the exhaust sample featured the lowest circularity value at 0.72. Convexity measures the smoothness of a particle’s contour, with higher values indicating fewer surface roughness and protrusions [65]. The <20 μm sample showed the highest value (0.95), while all other samples, except the exhaust sample, displayed similar convexity values. The exhaust sample exhibited the lowest convexity (0.90), indicating a rougher and more irregular morphology, as also observed in the micrographs (Figure 3d). The PSDs of the build plate and dispenser powder samples were similar, while overflow and exhaust powder samples exhibited broader PSDs. The overflow sample, characterized by a higher D90 value (73.1 μm), contained larger, partially melted, and agglomerated particles, likely originating from recoater-driven redistribution during printing. The exhaust sample exhibited the largest particle sizes and the widest PSD (D90 = 116.3 μm), indicative of heat-affected and re-solidified particles formed during the LPBF process. These findings align with previous studies on powder degradation in additive manufacturing, emphasizing the role of recoater dynamics and thermal exposure in altering powder morphology [66,67]. The build plate sample exhibited a narrower PSD and higher circularity, reflecting the predominance of unused virgin particles, whereas the overflow powder was comprised of heat-affected but partially melted particles [68]. The virgin < 20 μm powder sample exhibited the smallest PSD range (12.4–23.6 μm) in comparison with the PSDs of the other powder samples, due to the sieving process. While most particles in the build plate, overflow, exhaust, and dispenser samples exhibited mean diameters larger than typical A549 lung epithelial cell dimensions (24–42 μm), the sieved < 20 μm powder fraction was included to reflect respirable particle sizes relevant for pulmonary exposure. Although quantification of particles below 5 μm was not performed, fine particles below 20 μm were present at low proportions within the <20 μm sample (D10 = 12.4 μm). Furthermore, while particle internalization is generally size dependent, the cytotoxicity and oxidative stress responses observed, particularly in the sieved < 20 μm and exhaust powder samples, may also be attributed to extracellular mechanisms. These include surface oxidation-induced ROS generation and metal ion leaching, which can impair cellular function without the need for particle internalization. For clarity, all physicochemical and biological response data, including particle morphology, size distribution, cell viability, and oxidative stress, are summarized in Table 2.

**Figure 10:**
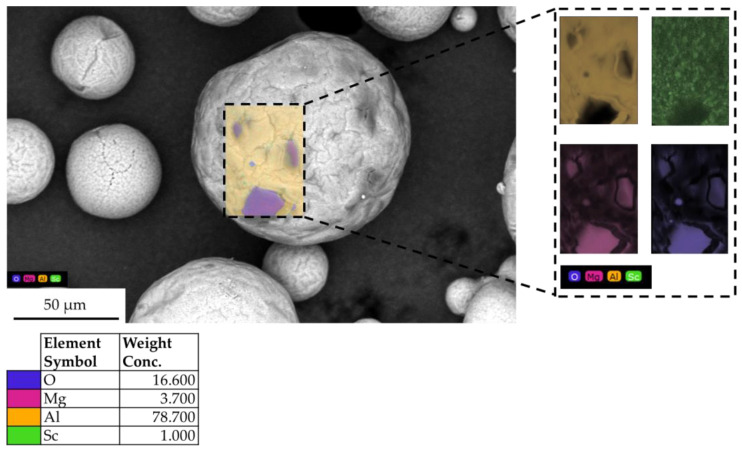
Indicative EDS elemental map chemical analysis on a heavily oxidized particle from the exhaust powder sample.

According to the EDS results (Figure 4), oxidation levels varied among the samples, with the highest oxygen content measured in the exhaust (10.9 wt.%) and overflow (10.0 wt.%) powders via EDS. This increase in oxygen concentration can be attributed to prolonged exposure to high temperatures in the LPBF process for both exhaust and overflow powder samples, leading to oxidation. Magnesium loss was also observed, as confirmed based on previous studies [69]. The slight increase in oxygen content in the virgin <20 μm sample compared to the dispenser sample is likely a result of increased surface area and exposure to ambient conditions (25 °C and 50% relative humidity) during handling and sieving [70,71,72]. Further EDS elemental map analysis (Figure 10) confirmed localized oxidation, with particles exhibiting darker surface regions having significantly higher oxygen content, containing O (16.6 wt.%), Mg (3.7 wt.%), Al (78.7 wt.%), and Sc (1.0 wt.%). An increased concentration of magnesium was also observed in the dark oxidized regions, indicating magnesium oxides. In aluminum–magnesium alloys, magnesium preferentially oxidizes over aluminum, forming MgO and MgAl_2_O_4_ in the oxide scale. The addition of Mg prevents the formation of Al_2_O_3_, leading to Mg-based oxides instead [73].

In the present study, cellular biological responses to the powders varied based on particle characteristics. The MTT assay results demonstrated that cell viability remained high for most samples, except for the exhaust and sieved < 20 μm powder, which induced a significant reduction in cell viability (*p* < 0.05). The exhaust powder’s higher toxicity is consistent with prior findings on metal-based powders, where agglomerated and heat-affected particles enhance cellular interactions and cytotoxic effects [74]. This is also supported by research showing that irregularly shaped particles and agglomeration may increase cellular uptake and inflammatory responses, as particle geometry has been shown to influence cytotoxicity and immune activation [75]. Oxidative stress assessment identified sample-dependent ROS generation. The virgin < 20 μm exhibited low cell viability across all concentrations and the highest oxidative stress levels, particularly at 60 μg/mL, correlating to their finer particle size. This supports findings from previous studies demonstrating that sieved metal powder particles (<10 μm), such as stainless steel and iron particles, showed a higher extent of metal ion release in cellular environments (bio-dissolution) compared to non-sieved (>10 μm) powder particles, most probably due to their enhanced surface reactivity [30]. Also, exhaust samples presented high oxidative stress, at 60 μg/mL, due to morphological changes such as low-circularity particles and agglomerates (fused particles). Alijagic et al. [30] similarly reported that agglomerated particles tend to release higher amounts of reactive metal ions, oxidative stress, and cytotoxic responses in cellular environments. The increased ROS levels observed in the exhaust powder suggest that surface modifications enhance its biological reactivity. Additionally, as agglomerates may undergo partial de-agglomeration in biological media, leading to an increased presence of fine particles, future studies should assess particle size distributions after dispersion in cell culture conditions. The high oxygen content in the exhaust and overflow powder samples may have occurred due to the surface oxides formed during the process [76,77]. Nevertheless, studies have demonstrated that the toxicity of aluminum particles is largely influenced by their higher reactivity and solubility, which contribute to increased cytotoxicity compared to aluminum oxides such as Al_2_O_3_ and AlO(OH). The poor solubility of aluminum oxides limits the release of bioactive aluminum ions, resulting in minimal impact on alveolar macrophage function and a significantly lower risk of lung inflammation [78]. Another study has validated that oxidized metal powders (316L) have been shown to release significantly fewer metal ions under neutral pH conditions due to stable surface oxide layers, resulting in lower toxicity compared to non-oxidized powders or bulk alloys. The protective oxide layer limits dissolution under neutral pH conditions (e.g., 7.2–7.4) and enhances corrosion resistance, thereby reducing cellular damage. [48]. While MgO can offer biocompatibility and high bioavailability [79,80], in this case, it is present only in localized spots on the surface of the particles rather than covering their surface, as indicated by the elemental EDS chemical analysis in Figure 10. The oxidation observed in the exhaust powder does not uniformly passivate the surface. Instead, the combination of irregular particle morphology, localized oxidation, and possible MgO-rich zones likely contributes to enhanced ROS generation, which outweighs any potential protective effect of a uniform oxide layer. As a result, the high toxicity in the exhaust is primarily influenced by irregular particle morphology. On the contrary, it is reported that abnormal levels of Mg^2+^ in the body can trigger various diseases, and some nanotheranostics based on Mg^2+^ have shown initial effectiveness in treating cancer. For instance, MgO nanoparticles exerted a strong inhibitory effect on cancer cells through the production of ROS because of the release of Mg^2+^ or of ligands, as well as byproducts. The generated ROS further caused oxidative damage to DNA, protein denaturation, and lipid peroxidation, leading to eventual cell death. This evidence can justify the increased cytotoxicity when cells are treated with the exhaust sample that exhibits characteristic dark-colored surface spots corresponding to MgO, based on the elemental mapping conducted. This is also consistent with the ROS measurement results available when cells are treated with the post-incubation liquid of the same powder sample, where the soluble metal(loid) contents act as cytotoxic contaminants and contribute to cytotoxicity potency caused by generated ROS [81].

**Figure 11:**
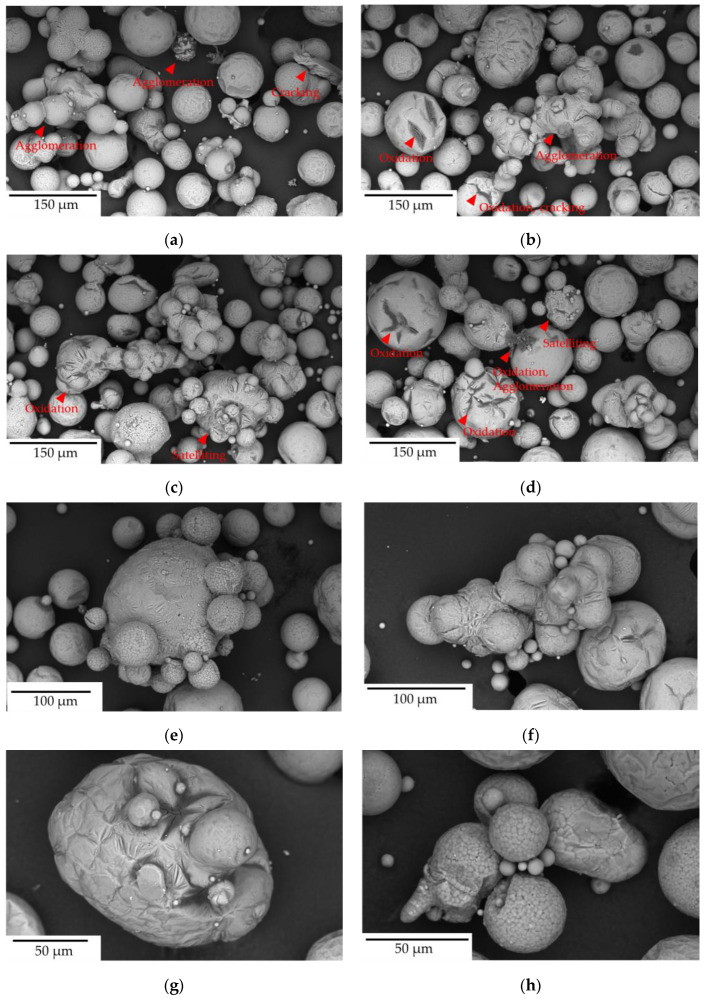

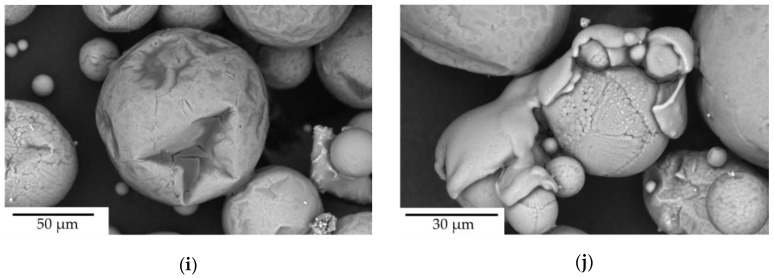
Scanning Electron Microscope (SEM) images of powder particles in an exhaust sample, highlighting various morphological defects: (**a**–**d**) Wide-field SEM images showing an overview of the powder sample, revealing a mixture of spherical, irregular, and agglomerated particles. Red arrows indicate key defects such as satelliting, oxidation, agglomeration, and cracking. (**e**) A satellited and agglomerated particle, where multiple smaller particles adhere to a larger core. (**f**) Agglomerated and elongated particles, formed due to partial fusion and sintering effects. (**g**) An oxidized particle with multiple satellite particles, exhibiting surface cracks and rough textures. (h) Satellited and oxidized particles with adhered smaller spheres and surface irregularities. (**i**) A sharp-edged and oxidized particle with a fractured surface. (**j**) Agglomerated and satellited particles exhibiting fused structures, areas of re-solidified melt, and attached smaller particles.

Although the overflow and exhaust samples exhibited comparable oxygen content, their cytotoxicity levels differed significantly, emphasizing that particle morphology has a higher impact on biological response than oxidation alone. The exhaust powder sample displayed low cell viability and high cytotoxicity, primarily due to its irregular, rough morphology and textured surface, as observed in the SEM images (Figure 11). This observation indicates that the powder is severely affected by the heat during the melting process. This phenomenon can occur when powder particles are situated close enough to a melt pool to be influenced by the heat, yet remain outside the part being formed. The extent to which the powder is affected is primarily determined by the duration of the build cycle, the number of parts, as well as their geometry and size. It is common to observe some degree of agglomeration and/or sintering, resulting in a few larger particles that occasionally exhibit satellites and a higher surface roughness. An important consideration is the spattering of ejected particles from the melt pool, which subsequently land in the surrounding powder. This typically results in the formation of agglomerates and oxides on the powder bed at a distance from the melt pool [82]. These morphological modifications, induced by high thermal exposure during LPBF, result in irregular and rough particle surfaces, which increase the geometric surface area and potentially enhance particle reactivity, promoting greater cellular interactions and oxidative stress responses. Studies have shown that irregularly shaped particles exhibit a higher surface area compared to round and smooth particles with the same particle size distribution and chemistry [83,84].

It can be also hypothesized that the cytotoxicities on A549 cells treated with the exhaust sample might be associated with the contents of water-soluble metal(loid)s, which might contribute an important proportion of the cytotoxicity potency of the aqueous extracts. The powder–aqueous extracts contain soluble metals involved in hydroxyl radical production in abiotic conditions. Water-soluble components activate ROS and release inflammatory cytokines and are also associated with cell apoptosis and genetic toxicity. Apoptotic volume decrease (AVD) in A549 cells exposed to the water-soluble fraction of particulate matter [50,85].

**Figure 12:**
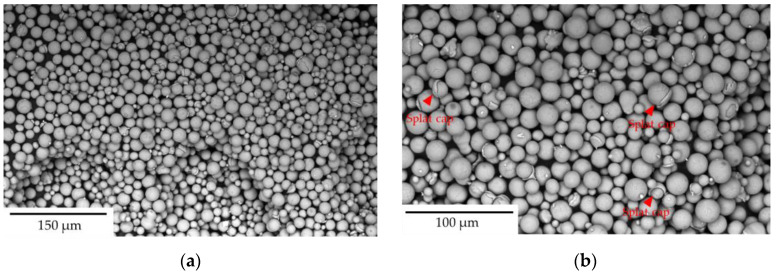
Scanning Electron Microscope (SEM) images of virgin powder particles (sieved < 20 µm), illustrating their morphological characteristics: (**a**,**b**) High-resolution images depicting the fine and predominantly smooth spherical particles characteristic of the sieved < 20 µm powder. The only notable morphological feature is the presence of splat caps on some particles, which are highlighted with red arrows, and which result from the atomization process.

In contrast, the sieved < 20 μm powder, despite its smooth and predominantly spherical morphology (Figure 12a), also exhibited reduced cell viability, though for a different reason—its small particle size. The increased surface-area-to-volume ratio of these finer particles enhances their reactivity and potential cellular uptake, making them more biologically active [86,87,88]. A distinctive feature observed in the sieved < 20 μm powder is the presence of splat caps, as highlighted in Figure 12b. These splat caps form during the atomization process when two particles collide just below the liquidus temperature when small hot droplets collapse onto a larger particle, resulting in cap formation [89,90]. Even if the virgin powder (<20 μm) contains splat caps, their shape remains mainly circular, and they retain their smooth surface, revealing that the size is the predominant factor affecting toxicity in this sample. This difference highlights the distinct pathways through which these two powder fractions develop cytotoxic effects—morphological degradation in the exhaust sample and small particle size in the sieved < 20 μm fraction. These findings align with previous studies indicating that smaller metal particles are more likely to be biologically reactive due to their high surface-area-to-volume ratio [91], whereas irregularly shaped and agglomerated particles tend to induce negative immune responses due to increased surface roughness and cellular interactions [92].

Indirect cytotoxicity assessment via media incubation did not show noticeable toxicity across samples. The possible release fraction from powder-incubated media did not induce significant cytotoxic effects in A549 cells, suggesting that the concentration of soluble metal ions released from the samples under the tested conditions was not sufficient to cause acute toxicity, despite the pronounced morphological and chemical variations across the samples, as presented in previous sections. Nevertheless, the exhaust (<20 um), overflow, and dispenser samples present a significant difference with respect to the CTRL sample (*p* value < 0.01), suggesting oxidative stress and a ROS level increase, resulting in an imbalance between antioxidants and reactive oxygen species (ROS). At this stage, and prior to cell death, cells respond to oxidative stress by activating or silencing genes that encode defensive enzymes, transcription factors, and structural proteins in order to restore redox balance [93].

Cell morphology assessment through SEM supported these observations (Figure 9). Control cells showed normal morphology, whereas cells exposed to virgin < 20 μm and exhaust powders exhibited increased surface roughness and membrane irregularities, which is indicative of mild to moderate oxidative stress responses. The exhaust sample caused the most pronounced morphological changes, including cytoskeletal disruptions and membrane damage due to the powder’s low shape descriptor and high PSD values. These findings highlight the variable effects of Scalmalloy powder samples on cell viability and emphasize the importance of controlling powder size and morphology to mitigate potential cytotoxicity.

The abovementioned powder characteristics, including oxygen contamination and agglomeration phenomena, are closely related to powder reuse considerations in LPBF. Such studies should consider powder degradation, oxide accumulation, and its effects on both material performance and biological safety. Beal et al. [32] reported that despite increased oxidation in reused powders, cytotoxicity and inflammatory responses remained stable across reuse cycles, suggesting that oxidation alone may not significantly impact toxicity. However, oxidation may influence mechanical properties, requiring controlled storage and handling to mitigate degradation. To enhance powder reusability and maintain its quality, it is essential to minimize exposure to oxygen by implementing controlled sieving conditions and improved powder handling.

## 5. Conclusions

This study provides an evaluation of the physicochemical and toxicological properties of Scalmalloy powders generated during LPBF processes, focusing on oxidation, particle size, and morphology, and their potential impact on biological responses. The findings demonstrate that low circular and irregular shapes and finer particle sizes influence cytotoxicity and oxidative stress, factors that are critical for both material performance and occupational safety. The exhaust and fine virgin powders (<20 µm) exhibited the lowest circularity (~0.72) and particle size (~15 μm) correspondingly, and most pronounced cytotoxic effects (~60 to 69% viability), correlated with increased oxidative stress (*p* < 0.01). In contrast, dispenser, overflow, and build plate powders retained more uniform morphology and larger particle sizes, and demonstrated higher cell viability (>85%). The in vitro experiments demonstrated that direct particle exposure led to higher oxidative stress and cytotoxicity in finer powder fractions (<20 μm) and more irregular and agglomerated powders (exhaust). Morphological characteristics such as size and shape, combined with localized and non-uniform oxidation, have a higher impact on toxicity than the increased oxygen content. These findings emphasize the importance of controlling powder morphology and size distribution to minimize potential health risks associated with LPBF powder exposure. Indirect exposure via media incubation resulted in non-significant toxic effects, suggesting minimal metal dissolution and ion release under the specific conditions, yet noticeable ROS generation due to cell oxidative stress.

These findings contribute to a better understanding of how oxidation and particle characteristics influence the biological impact of LPBF Scalmalloy powder, providing essential insights for safer handling and exposure risk mitigation in additive manufacturing environments. Future research should investigate long-term exposure effects, quantification of metal ion release, and potential risks of skin sensitization due to dermal contact with powder fractions. Finally, oxidative stress markers should be investigated and correlated to gene expression changes in order to comprehensively assess biocompatibility for applications in additive manufacturing.

## Figures and Tables

**Table 1:** Nominal chemical composition of Scalmalloy^®®^ based on the provider’s powder specification datasheet.

**Chemical Composition (%)**	**Element**	**Mg**	**Sc**	**Zr**	**Mn**	**Fe**	**Si**	**Ti**	**P**	**Ni**	**O**	**H**	**Others**	**Al**
	min	4.20	0.68	0.20	0.30	-	-	-	-	-	-	-	NA	Balance
	max	5.10	0.88	0.50	0.80	0.40	0.40	0.15	0.03	0.03	0.10	0.05	NA	

**Table 2:** Physicochemical properties and biological responses of Scalmalloy powder samples, highlighting the impact of powder morphology and oxidation levels on cytotoxicity and oxidative stress. Direct exposure represents samples upon treatment with Scalmalloy powder in various concentrations, while indirect exposure represents samples upon treatment with media post-incubation with Scalmalloy powder. The table presents particle size distribution (PSD), circularity, convexity, oxidation levels, and biological responses.

Samples	PSD (D10, D50, D90) (μm)	Mean Circle Equivalent Diameter (μm)	Circularity	Convexity	Oxidation Levels (wt.%)	Cell Viability (%) (60 μg/μL)	Oxidative Stress (60 μg/μL)	Cell Viability (%) (60 μg/μL)	Oxidative Stress (60 μg/μL)
						Direct Analysis		Indirect Analysis	
Virgin < 20 μm	12.4 ± 0.3, 18.2 ± 0.9, 23.6 ± 1.5	15.0 ± 0.3	0.86 ± 0.01	0.95 ± 0.01	7.1	60.68	1.42	86.35	1.16
Dispenser	32.5 ± 0.9, 50.9 ± 0.5, 68.6 ± 0.6	33.8 ± 1.7	0.76 ± 0.02	0.91 ± 0.01	6.0	85.78	1.21	90.18	1.33
Overflow	33.1 ± 0.2, 53.4 ± 0.4, 73.1 ± 3.3	31.5 ± 0.2	0.75 ± 0.01	0.91 ± 0.01	10.0	87.79	1.33	88.43	1.33
Exhaust	44.6 ± 3.8, 71.4 ± 8.9, 116.3 ± 29.1	41.8 ± 2.3	0.72 ± 0.01	0.90 ± 0.01	10.9	69.48	1.42	89.87	1.26
Build Plate	24.3 ± 0.9, 40.4 ± 0.9, 58.6 ± 0.2	24.4 ± 0.9	0.77 ± 0.01	0.92 ± 0.01	7.1	94.98	1.25	86.74	1.16

## Data Availability

The data are included in the article and are available on request from the corresponding author.

## Abbreviations

AM	Additive Manufacturing
LPBF	Laser Powder Bed Fusion
PSD	Particle Size Distribution
SEM	Scanning Electron Microscopy
EDS	Electron Dispersive Spectroscopy
FOV	Field of View
ROS	Reactive Oxygen Species
DMEM	Dulbecco’s Modified Eagle Medium
FBS	Fetal Bovine Serum
DMSO	Dimethyl Sulfoxide
PBS	Phosphate-Buffered Saline
PFA	Paraformaldehyde
HMDS	Hexamethyldisilazane
SED	Secondary Electron Detector
MTT	3-(4,5-Dimethylthiazol-2-yl)-2,5-diphenyltetrazolium bromide
ATCC	American Type Culture Collection
ANOVA	Analysis of Variance
RPM	Revolutions Per Minute
μm	Micrometer
wt.%	Weight Percentage
Cr6+	Hexavalent Chromium
MgO	Magnesium Oxide
Al_2_O_3_	Aluminum Oxide
AlO(OH)	Aluminum Oxyhydroxide
TBHP	Tert-butyl hydroperoxide
DCFDA	Dichlorofluorescin diacetate
DCF	Dichlorofluorescein
DCFH	Dichlorofluorescin
AVD	Apoptotic volume decrease
HO•	Hydroxyl radicals
O2•−	Superoxide
H2O2	Hydrogen peroxide
NADPH	Nicotinamide adenine dinucleotide phosphate
